# Structural Dynamics of the N-Extension of Cardiac Troponin I Complexed with Troponin C by Site-Directed Spin Labeling Electron Paramagnetic Resonance

**DOI:** 10.1038/s41598-019-51740-6

**Published:** 2019-10-24

**Authors:** Chenchao Zhao, Takayasu Somiya, Shinji Takai, Shoji Ueki, Toshiaki Arata

**Affiliations:** 1 Department of Biological Sciences, Graduate School of Science, Osaka University, Machikaneyama-cho 1-1, Toyonaka, Osaka 560-0043 Japan; 2 Center for Advanced High Magnetic Field Science, Graduate School of Science, Osaka University, Machikaneyama-cho 1-1, Toyonaka, Osaka 560-0043 Japan; 3 Kagawa School of Pharmaceutical Sciences, Tokushima Bunri University, Shido 1314-1, Samuki, Kagawa 769-2193 Japan; 4 Present Address: Department of Biology, Graduate School of Science, Osaka City University, Sugimoto 3-3-138, Osaka, Osaka 558-8585 Japan

**Keywords:** Biological sciences, Intrinsically disordered proteins, Molecular biophysics, Muscle contraction

## Abstract

The secondary structure of the N-extension of cardiac troponin I (cTnI) was determined by measuring the distance distribution between spin labels attached to the *i* and *i* + 4 residues: 15/19, 23/27, 27/31, 35/39, and 43/47. All of the EPR spectra of these regions in the monomeric state were broadened and had a amplitude that was reduced by two-thirds of that of the single spin-labeled spectra and was fit by two residual distance distributions, with a major distribution one spreading over the range from 1 to 2.5 nm and the other minor peak at 0.9 nm. Only slight or no obvious changes were observed when the extension was bound to cTnC in the cTnI-cTnC complex at 0.2 M KCl. However, at 0.1 M KCl, residues 43/47, located at the PKC phosphorylation sites Ser42/44 on the boundary of the extension, exclusively exhibited a 0.9 nm peak, as expected from α-helix in the crystal structure, in the complex. Furthermore, 23/27, which is located on the PKA phosphorylation sites Ser23/24, showed that the major distribution was markedly narrowed, centered at 1.4 nm and 0.5 nm wide, accompanying the spin label immobilization of residue 27. Residues 35 and 69 at site 1 and 2 of cTnC exhibited partial immobilization of the attached spin labels upon complex formation. The results show that the extension exhibited a primarily partially folded or unfolded structure equilibrated with a transiently formed α-helix-like short structure over the length. We hypothesize that the structure binds at least near sites 1 and 2 of cTnC and that the specific secondary structure of the extension on cTnC becomes uncovered when decreasing the ionic strength demonstrating that only the phosphorylation regions of cTnI interact stereospecifically with cTnC.

## Introduction

In both skeletal and cardiac muscle, contraction is regulated by troponin (Tn). To respond to the initial signal of an increased Ca^2+^ concentration, the hydrophobic pocket of the N-domain of TnC opens by Ca^2+^ binding and attracts the regulatory region of TnI, followed by the departure of the inhibitory region of TnI from its original repressive site to allosterically modulate tropomyosin leaving the potential actin and myosin interactive site to initiate the cross-bridge cycle^1–4^. However, in contrast to skeletal TnI, cardiac TnI (cTnI) possesses a unique N-extension (1–32 amino acids) that does not have a crystal structure^5^. This extension is functional for sufficient opening of the cTnC N-domain^6–8^ as well as maintaining the normal Ca^2+^ sensitivity of cTnC^9–12^. Furthermore, phosphorylation sites (23/24 and 42/44) for protein kinase A (PKA) and C (PKC) are present at or adjacent to this extension and modulate the Ca^2+^ sensitivity after phosphorylation^4,13^. Despite a large number of studies by NMR, small-angle X-ray scattering, fluorescence energy transfer spectroscopy or chemical cross-linking attempting to elucidate structural information about the intrinsically disordered region (IDR) cTnI N-extension^14–20^, the detailed conformation and the interaction between the extension and cTnC are still unknown.

Recently, continuous wave (CW)-EPR and pulsed electron double resonance (PELDOR or DEER) spectroscopy have been used to investigate the interspin distance between intra- and intersubunit sites in skeletal ternary TnC-I-T or cardiac binary TnC-I complexes and in reconstituted muscle fibers where the specific residues are mutated and spin labeled^21–24^. These studies have verified the structures of the cTnC monomer^22,23^ and the binary cTnC-cTnI complex^21,24^ derived from X-ray crystallography and NMR studies and determined the Ca^2+^-induced conformational transition of TnC in the binary complex and in fibers^22,23^. Furthermore, we found a large Ca^2+^-dependent distance change between Cys133 in skeletal TnI and several residues in skeletal TnC in the TnC-I-T complex or in reconstituted thin filaments using PELDOR^24^. Here, we applied these techniques to investigate the structure and dynamics of cTnI’s N-extension. Based on the fact that this extension retained the ability to bind cTnC and Ca^2+^, as well as the phosphorylation-dependent regulation of actomyosin ATPase after site-directed mutagenesis^17–20,25^, the secondary structure of the N-extension of cTnI complexed with cTnC was determined by measuring the distance distribution between spin labels attached to the *i* and *i* + 4 residues. All of the EPR spectra broadened, and the residual distance exhibited a broad distribution over 1–2 nm or more and a sharp peak at 0.9 nm, suggesting mainly partially folded or complete unfolded structure equilibrated with a partial α-helix-like structure. Only slight or no obvious changes were observed when the extension was bound to cTnC in the cTnI-cTnC complex, as previously reported for characteristics of IDR during interaction with other proteins^26^. However, the specific secondary structure of the extension on cTnC could be uncovered by lowering the ionic strength. In particular, the 23/27 and 43/47 pairs showed a more broadened spectra in the cTnI-cTnC complex than in the monomer. Distance analysis showed that the 23/27 and 43/47 pairs had phosphorylation sites Ser23/24 and 42/44, and transformed from flexible in the monomeric state to a relatively stable and extended conformation in the cTnI-cTnC complex. The residues 35 and 69 at site 1 and 2 of cTnC showed immobilization of attached spin labels in the complex, respectively. These results suggest that the N-extension interacts with cTnC at least near site 1 and site 2 and that the extension of cTnI is mostly disordered but partially folded, whereas PKA and PKC sites represent more stable conformations.

## Results and Discussion

### Interresidual distance of the spin-labeled N-extension of cTnI

**Figure 1:**
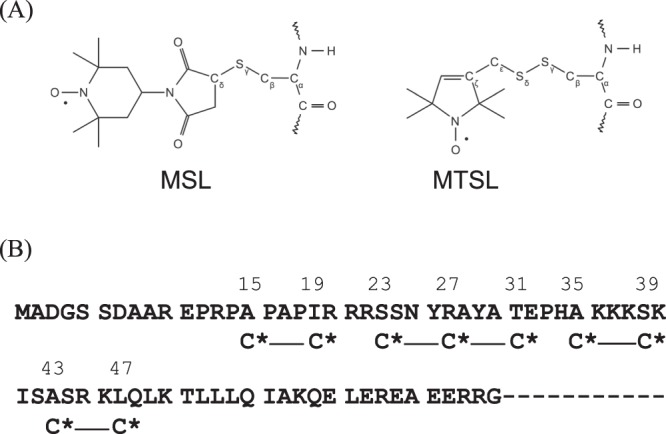
(**A**) Structures of the spin labels used in this study, with the cysteine disulfide bond: 4-maleimido-2,2,6,6-tetramethyl-1-piperidinyloxy (MSL); (1-oxyl-2,2,5,5-tetramethylpyrrolidin-3-yl) methyl methanethiosulfonate (MTSL). (**B**) Position of spin-labeled residues (C*) in the amino acid sequence of the cysteine mutants of cTnI.

**Figure 2:**
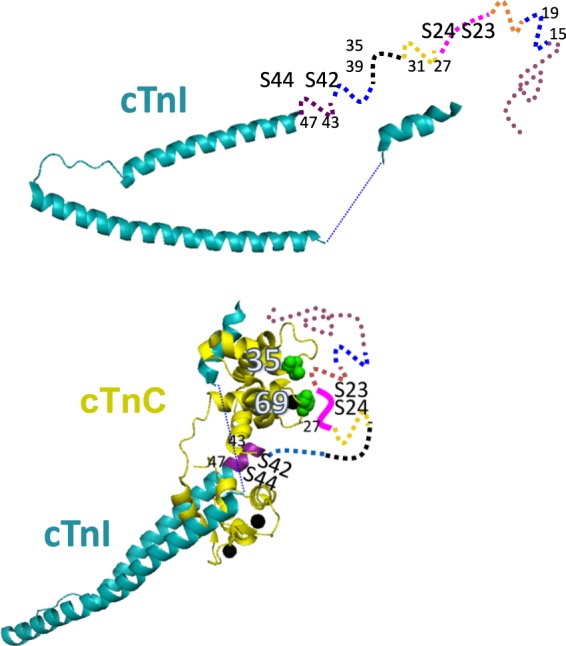
Proposed conformations of the N-extension of cTnI in the monomeric and in the dimeric complex. The extension exhibited mainly partial folding at residues 15/19, 27/31 and 43/47 or completely unfolded structure at residues 23/27 and 35/39 equilibrated with a minor α-helix component over the length in the monomeric state. The minor α-helix is not shown to avoid complexity. The extension binds to the N-lobe of cTnC near residues 35 and 69 at site 1 and 2, as suggested from the immobilization of the spin label attached to these residues of cTnC and residue 27 of the extension. Further, residues 23/27 including phosphorylatable site S23/24 became a stable extended conformation and residues 43/47 including the other site S42/44 forms an α-helix. The structure of the binary cTnC-cTnI complex is extracted from that of the ternary cTnC-I-T2 complex (PDB code 1J1D).

**Figure 3:**
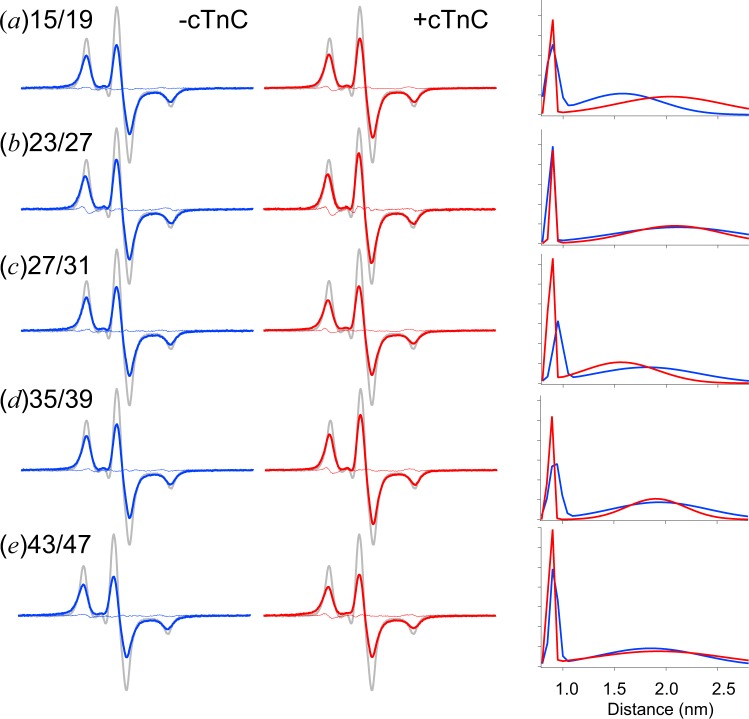
Distance analysis of the EPR spectra from the double MSL-labeled cTnI mutant with and without cTnC at high ionic strength (0.2 M KCl). The EPR spectra were obtained at 170 K. Spectra from cTnI mutants labeled at residues 15/19 (**a**), 23/27 (**b**), 27/31 (**c**), 35/39 (**d**), and 43/47 (**e**) were obtained in the absence (*blue line in the left panel*) and presence (*red line in the central panel*) of cTnC. The scan width was 200 G. For each panel, the experimental spectrum of the double-labeled cTnI mutant (*colored line*) was compared with the spectrum of the single-labeled mutant (*gray line*) normalized to the same number of spins. The experimental spectrum with or without cTnC was fitted by two Gaussian distance distributions shown by the red or blue line in the right panel, respectively. Residual from the best-fit spectrum is shown in the left or central panels (*thin colored line*).

It would be interesting to determine whether the N-terminal extension of cTnI is unstructured or maintains its secondary structures and whether it changes structures after complex formation of cTnI with cTnC. To examine these possibilities, two nitroxide-labeled double-cysteine mutants at the *i* and *i* + 4 residues were prepared, and the relative distances between cysteine residues were measured *via* EPR spectroscopy at low temperature (170 K) as done previously for secondary structures and dynamics of loop regions^27^. From previous studies by using NMR or cross-linking, 15–30 amino acids in the cTnI N-extension were restricted to be a functional candidate region to bind to cTnC’s N-domain, and the first 15 amino acids turned out to be fluttering around and had little effect on the Ca^2+^ sensitivity of the actomyosin ATPase rate after site-directed mutagenesis^17–20,25^. Then, the two residues were mutated to cysteine and spin labeled with 4-maleimido-2,2,6,6-tetramethyl-1-piperidinyloxy (MSL) (Fig. 1). We focused on the four putative cTnC binding regions 15/19, 23/27, 27/31, 35/39, and 43/47 in the boundary of the IDR that had PKC phosphorylation sites (Fig. 2). Figure 3 shows the double labeled spectra, which were measured at 0.2 M KCl. The spectra appeared to be broadened, and their amplitude measured either with or without cTnC descended to approximately two-thirds of that of the single labeled spectra (MSL at residue 20), which indicated the presence of an electron dipolar interaction. We found that all showed a broadened distribution over 0.8–2.5 nm with a center distance of 1.5–2.3 nm and a 2 nm width from single Gaussian fitting (Table 1). We could also analyze the data by using 2 Gaussian distributions with 1.2–2.6 times better χ^2^, and a small fraction (~20%) had a sharp distribution centered at 0.9 nm with a 0.05–0.1 nm width (Fig. 3 *right*; Table 1), and a large fraction (~50%) had a very broad distribution ranging from 0.8 nm to 2.5 nm (centered at 1.5–2 nm with a 1–1.5-nm width). For some cases, the fitting was poor, as evaluated by the scatter of residuals and χ^2^ (Table 1). The poor fitting is likely to be due to significant exchange coupling, which causes spectral narrowing^28^. The distance of 0.9 nm is similar to that of a model α-helical peptide^28,29^. The results demonstrate that all entire regions of N-extension, even 43/47, which has a crystal structure that is available in part, exhibit a mixture or equilibrium between a largely melted main structure and a partially folded α-helix-like structure. There was very small or no difference in the broad distance distributions of the monomer and complex states. Only spin labels at residues 43 and 47 showed small but discernable mobility changes to the slow component at room temperature when complexed with cTnC. However, the other residues, namely, 15/19, 23/27, 27/31, and 35/39, have no or slight mobility changes with cTnC (data not shown). These no or slight structural changes are very much consistent with that previously reported for characterization of spin-labeled IDR during the interaction with a target protein^26^.

**Table 1:** Distance distributions between the two spin labels attached at *i* and *i* + *4* residues in the N-extension of cTnI with and without cTnC at 0.2 M KCl.

KCl (M)	Protein	Number of Gaussians	χ^2^	*r* (nm)	∆*r* (nm)	*f* (%)
0.2	cTnI(15/19MSL)	1	5.6	1.73 ± 0.04^a^ > 2.5	2.0 ± 0.3^a^	70 30 ± 2
		2	3.7	0.89 ± 0.01 1.58 ± 0.02 > 2.5	0.13 ± 0.01 0.90 ± 0.09	23 ± 3 49 27 ± 2
	cTnI(15/19MSL) + cTnC	1	18	2.09 ± 0.03 > 2.5	2 ± 0.4	70 30 ± 3
		2	12	0.88 ± 0.01 2.04 ± 0.07 > 2.5	0.05 ± 0.04 1.2 ± 0.2	18 ± 3 55 27 ± 3
	cTnI(23/27MSL)	1	8.8	2.01 ± 0.17 > 2.5	2 ± 0.5	68 32 ± 4
		2	7.7	0.88 ± 0.02 2.12 ± 0.17 > 2.5	0.05 ± 0.06 1.5 ± 0.4	18 ± 5 56 26 ± 4
	cTnI(23/27MSL) + cTnC	1	9.0	2.22 ± 0.36 > 2.5	2 ± 0.7	60 40 ± 5
		2	7.9	0.90 ± 0.03 2.09 ± 0.13 > 2.5	0.05 ± 0.06 1.1 ± 0.3	12 ± 6 49 39 ± 5
	cTnI(27/31MSL)	1	3.1	1.56 ± 0.04 > 2.5	2 ± 0.3	65 35 ± 2
		2	2.5	0.95 ± 0.01 1.81 ± 0.03 > 2.5	0.10 ± 0.02 1.2 ± 0.2	16 ± 2 49 35 ± 2
	cTnI(27/31MSL) + cTnC	1	1.8	1.75 ± 0.05 > 2.5	2 ± 0.4	71 29 ± 2
		2	1.4	0.88 ± 0.01 1.56 ± 0.02 > 2.5	0.05 ± 0.05 0.8 ± 0.1	24 ± 5 47 29 ± 2
	cTnI(35/39MSL)	1	5.8	1.79 ± 0.05 > 2.5	2 ± 0.3	66 34 ± 2
		2	3.6	0.93 ± 0.01 1.94 ± 0.04 > 2.5	0.12 ± 0.02 1.1 ± 0.2	19 ± 2 49 32 ± 2
	cTnI(35/39MSL) + cTnC	1	14	2.26 ± 0.29 > 2.5	2 ± 0.6	61 39 ± 4
		2	7.9	0.88 ± 0.02 1.91 ± 0.05 > 2.5	0.05 ± 0.04 0.7 ± 0.1	18 ± 3 37 45 ± 2
	cTnI(43/47MSL)	1	6.4	1.59 ± 0.04 > 2.5	2 ± 0.3	75 25 ± 2
		2	3.3	0.91 ± 0.01 1.85 ± 0.03 > 2.5	0.08 ± 0.01 1.2 ± 0.2	23 ± 2 55 22 ± 2
	cTnI(43/47MSL) + cTnC	1	14	1.54 ± 0.06 > 2.5	2 ± 0.4	71 29 ± 3
		2	5.4	0.88 ± 0.01 1.93 ± 0.07 > 2.5	0.05 ± 0.03 1.5 ± 0.3	25 ± 3 54 21 ± 2

*r* is the central distance, *Δr* is the width of the distance distribution, and *f* is the percentage contribution of the Gaussian population or non-interacting spins.

^a^Indicates a best estimate ± uncertainty.

It was inferred that the distance distribution is partially dependent on the ionic strength and pH for the IDR of proteins because N-extension contains many charged residues, although in some cases, IDR folding dynamics are independent of ionic strength^30^. Additionally, the binding of the cTnI extension to cTnC depends on the ionic strength due to its charged residues and is weaker at higher ionic strength. In fact, the calorimetric study showed that the binding of cTnI to the N-terminal domain of cTnC is strongly dependent on the ionic strength^31^. On the other hand, spin-labeled MSL is more dynamic (covers a larger protein surface or peptide chain by possible rotamers) than (1-oxyl-2,2,5,5-tetramethylpyrrolidin-3-yl) methyl methanethiosulfonate (MTSL), and the distance between C_β_ and the N-O bond for each spin label in a completely extended conformation is as much as 0.91 and 0.77 nm for MSL and MTSL, respectively (Fig. 1). In fact, the distance distribution for MSL was reported to be broader by 0.5–0.6 nm in width than that for MTSL^32^. However, we do not expect MTSL can detect any changes in the distance and mobility with/without cTnC at high ionic strength, because almost complete disorder and no or slight mobility change of MSL with/without cTnC at high ionic strength. To uncover the specific secondary structure of the N-extension by its strong interaction with cTnC and by the reduced fluctuation of the attached spin-label rotamer, the ionic strength was decreased to approximately half (0.1 M KCl), and the MSL spin label was substituted by MTSL.

**Figure 4:**
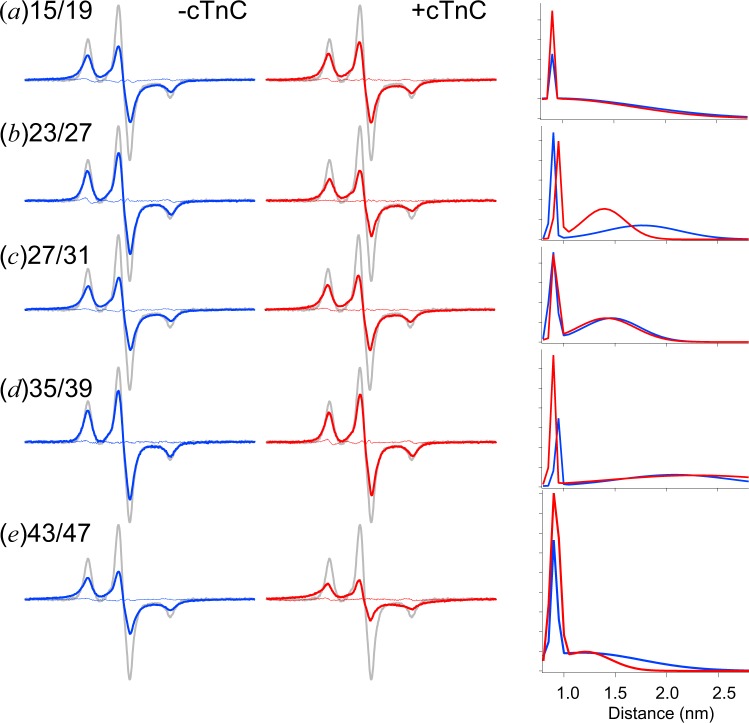
Distance analysis of the EPR spectra from the double MTSL-labeled cTnI mutant with and without cTnC at low ionic strength (0.1 M KCl). Spectra from cTnI mutants labeled at residues 15/19 (**a**), 23/27 (**b**), 27/31 (**c**), 35/39 (**d**), and 43/47 (**e**) were obtained in the absence (*blue line in the left panel*) and presence (*red line in the central pane*l) of cTnC. For each panel, the experimental spectrum (*colored line*) was compared with the spectrum of the single-labeled mutant (*gray line*). In the right panel, two Gaussian distance distributions are displayed for cTnI with (*red line*) or without (*blue line*) cTnC. Other conditions were as described in Fig. 3.

**Figure 5:**
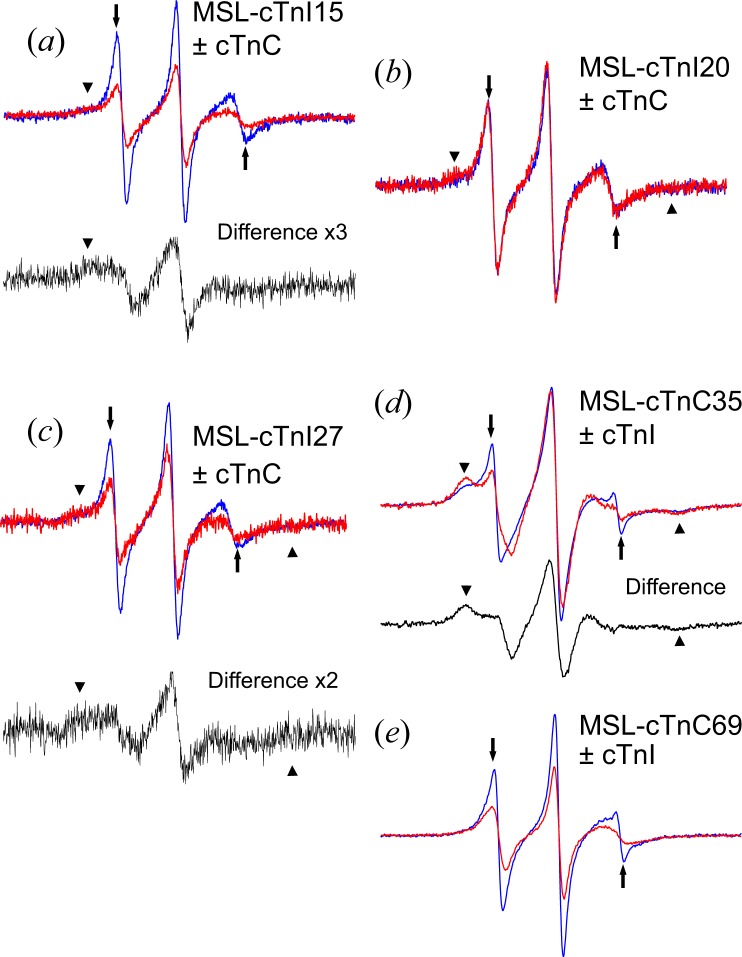
Overlay of the EPR spectra from the MSL-labeled cTnI with and without cTnC (**a**–**c**) and from the MSL-labeled cTnC mutants with and without cTnI (**d**,**e**). The spectra shown in the blue and red lines were taken from the MSL-cTnI or MSL-cTnC alone and the MSL-cTnI-cTnC or MSL-cTnC-cTnI complex, respectively. The black lines show the difference spectra produced by subtracting the MSL-cTnI or MSL-cTnC spectra from the MSL-cTnI-cTnC or MSL-cTnC-cTnI spectra, respectively (**a**,**c**,**d**). (**a**) Spectra from cTnI labeled at residue 15 with MSL. The difference spectrum was produced by multiplying 3-fold after subtracting 34% of the MSL-cTnI spectrum from the MSL-cTnI-cTnC spectrum. (**b**) Spectra from cTnI at residue 20 with MSL. The difference spectrum was produced by multiplying 2-fold after subtracting 40% of the MSL-cTnI spectrum from the MSL-cTnI-cTnC spectrum. (**c**) Spectra from cTnI at residue 27 with MSL (**d**) Spectra from cTnC labeled at residue 35 with MSL. The difference spectrum was produced by subtracting 45% of the MSL-cTnC spectrum from the MSL-cTnC-cTnI spectrum. (**e**) Spectra from cTnC labeled at residue 69. The peaks from fast and slow components are indicated by the arrows and arrowheads, respectively. All spectra were measured at 0.1 M KCl. The scan width was 100 G.

As shown in Fig. 4 (*left panels, blue lines)*, for the cTnI full-length monomer at 0.1 M KCl, the double labeled spectra appeared to be broadened, and their amplitude decreased to approximately three fourths to one half of that of the single labeled spectra (MTSL at residue 20, *gray lines*), which indicates the presence of an electron dipolar interaction. The distance distribution was obtained by simulating and fitting double labeled spectra from the single spin-labeled cTnI R20C, as described in the Materials and Methods (Table 2). Obviously, the spectra for residues 15/19, 27/31 and 43/47, except for the boundaries among the three peaks, became convergent (Fig. 4a,c,e, *left panels, blue lines)*. According to the simulation obtained from spectral fitting by using a single Gaussian distribution, for the cTnI monomer, the distance shortened (center distance of 0.8 nm) but still had a relatively broad distribution (full width of 1.5–2 nm) (Table 2). The spectrum for 23/27 or 35/39 had a slightly decreased amplitude (Fig. 4b,d) and recovered a broad longer distance distribution centered at 2.8 nm with a 2-nm width or with >2.5 nm population (beyond sensitivity), respectively (Table 2). We could also analyze the data by using 2 Gaussian distributions, which had a 1.2–1.9 times better χ^2^ for all with a small fraction (~15%) centered at 0.9 nm with 0.05-nm width (Fig. 4, *right panels*; Table 2). The distance of 0.9 nm appears identical to that observed at 0.2 M KCl. For residues 15/19, the 0.9 nm peak may not be significant because of the large uncertainty (Fig. 4a; Table 2). The mobility of the spin label at residue 15, 20 or 27 showed no indication of aggregates and only a single motional component (effective correlation times = 1–2 ns) that is so fast that it could not be resolved into α-helical and unfolded structures (Fig. 5a–c, *blue lines*). It is, therefore, likely that the small fraction with short and very narrow distance distribution at 0.9 nm comes from the partially or transiently folded α-helix-like short structure of the cTnI N-extension. The distance of a large fraction (40–50%) for 15/19 and 27/31, and 43/47 was shortened compared with 0.2 M KCl but still had a broad distribution ranging from 1 to 2 nm (centered at 0.9–1.4 nm with a 0.6–2-nm width) (Fig. 4a,c,e; Table 2). The centers of the broader distributions appeared smaller by ~0.4 nm than those at 0.2 M KCl. This may be because the length of MTSL is shorter than MSL causing the results to be dynamically disordered. The results demonstrated a largely melted helix conformation of the N-extension in the monomeric cTnI that is in equilibrium with the partially or transiently folded short α-helix-like structure. It could not be excluded that the melted helix structure includes an extended left-handed poly (L-proline) II helix. For 23/27 and 35/39, a small fraction (~15%) centered at 0.9 nm with a 0.05-nm width and a large fraction (30–40%) had a relatively broader distribution ranging from 1 nm to 2.5 nm (centered at 2 nm with a 1–1.3-nm width) (Fig. 4b,d, *right panels;* Table 2), suggesting that these regions include a completely melted and flexible structure together with transient α-helix-like short structure in the monomeric state.

**Table 2:** Distance distributions between the two spin labels attached at *i* and *i* + *4* residues in the N-extension of cTnI with and without cTnC at 0.1 M KCl.

Protein	Number of Gaussians	χ^2^	*r* (nm)	*Δr* (nm)	*f* (%)
cTnI(15/19MTSL)	1	4.8	1.00 ± 0.06^a^ > 2.5	2 ± 0.3^a^	67 33 ± 2^a^
	2	4.2	0.90 ± 0.03 0.91 ± 0.40 > 2.5	0.04 ± 0.38 2 ± 0.6	6 ± 60 61 33 ± 2
cTnI(15/19MTSL) + cTnC	1	2.4	0.8 ± 0.15 > 2.5	1.7 ± 0.2	67 33 ± 1
	2	1.6	0.90 ± 0.03 0.94 ± 0.25 > 2.5	0.04 ± 0.13 1.74 ± 0.35	11 ± 28 54 35 ± 1
cTnI(23/27MTSL)	1	4.3	0.8 ± 0.4 > 2.5	2 ± 0.6	43 57 ± 2
	2	3.0	0.89 ± 0.01 1.77 ± 0.04 > 2.5	0.05 ± 0.02 0.86 ± 0.18	16 ± 2 32 52 ± 2
cTnI(23/27MTSL) + cTnC	1	1.9	1.19 ± 0.02 > 2.5	1.02 ± 0.06	58 42 ± 1
	2	1.5	0.94 ± 0.01 1.40 ± 0.01 > 2.5	0.06 ± 0.02 0.48 ± 0.07	15 ± 2 40 45 ± 1
cTnI(27/31MTSL)	1	3.1	0.82 ± 0.19 > 2.5	2 ± 0.3	66 34 ± 1
	2	2.5	0.90 ± 0.01 1.47 ± 0.01 > 2.5	0.08 ± 0.01 0.65 ± 0.08	19 ± 2 42 39 ± 1
cTnI(27/31MTSL) + cTnC	1	1.8	0.8 ± 0.13 > 2.5	1.62 ± 0.17	63 37 ± 1
	2	1.4	0.92 ± 0.02 1.41 ± 0.02 > 2.5	0.05 ± 0.08 0.66 ± 0.08	21 ± 9 37 42 ± 1
cTnI(35/39MTSL)	1	2.4	2.76 ± 0.54 > 2.5	2 ± 0.6	65 35 ± 4
	2	1.5	0.94 ± 0.02 2.09 ± 0.12 > 2.5	0.05 ± 0.05 1.33 ± 0.28	10 ± 2 39 51 ± 3
cTnI(35/39MTSL) + cTnC	1	4.6	1.42 ± 0.13 > 2.5	2 ± 0.6	46 54 ± 3
	2	3.7	0.89 ± 0.04 2.28 ± 0.45 > 2.5	0.05 ± 0.06 1.99 ± 0.94	19 ± 8 44 37 ± 3
cTnI(43/47MTSL)	1	3.0	0.8 ± 0.12 > 2.5	1.54 ± 0.16	65 35 ± 1
	2	1.6	0.91 ± 0.02 1.18 ± 0.12 > 2.5	0.06 ± 0.01 1.34 ± 0.21	21 ± 4 50 29 ± 1
cTnI(43/47MTSL) + cTnC	1	6.4	0.8 ± 0.05	0.67 ± 0.06	75 25 ± 1
	2	2.3	0.91 ± 0.01 1.21 ± 0.07 > 2.5	0.10 ± 0.01 1.32 ± 0.28	48 ± 5 29 23 ± 1

*r* is the central distance, *Δr* is the width of the distance distribution, and *f* is the percentage contribution of the Gaussian population or non-interacting spins.

^a^Indicates a best estimate ± uncertainty.

In the complex state and during the process of coupling folding, the segments reside in a high-energy state to seek a suitable conformation and then collapse into that confirmation. However, among the five regions we examined, 15/19, 27/31 and 35/39 showed no significant change in spectra and distance distributions, indicating a partially folded or melted helix conformation (15/19, 27/31) and a completely melted conformation (35/39) in both the monomeric and cTnI-C complex state (Fig. 4a,c,d, *middle panels, red lines;* Table 2). Interestingly, 23/27 and 43/47 more strongly demonstrated broadened spectra in the cTnI-cTnC complex than in the monomer. The best-fit simulation showed that 23/27 containing PKA phosphorylation sites Ser23/24 has a discernable transition from flexible to a relatively stable extended conformation with a central distance (1.19 nm) and narrow width (1.02 nm) (Fig. 4b*, middle panels, red line;* Table 2). This is due to lowering the ionic strength but not to replacing MSL with MTSL, because the MTSL-labeled 23/27 with cTnC at 0.2 M KCl showed a slightly narrowed but still broad distribution (data not shown).

We could also analyze the data of the complex state by using 2 Gaussian distributions with 1.3–2.8 times better χ^2^ (Fig. 4, *right panel, red line*; Table 2). For residues 15/19, 23/27, 27/31 and 35/39, the 0.9 nm peak (10–20%) appeared as in the monomeric state (Fig. 4a–d; Table 2). This peak may not be significant for 15/19 because of the large uncertainty (Fig. 4a; Table 2). For residues 15/19, 27/31 and 35/39, a large fraction (40–50%) had a relatively broader distribution ranging from 1 to 1.5–2.5 nm (centered at 0.9–2 nm with a width of 0.6–2 nm) as in the monomeric state (Fig. 4a,c,d; Table 2). This result demonstrated these regions take a largely melted helix conformation of the N-extension even in the complex state that is in equilibrium with the partially or transiently folded short α-helix-like structure. On the other hand, for residues 23/27, a large fraction (40%) had a shortened and narrowed distribution ranging from 1.2 to 1.7 nm (centered at 1.4 nm with a width of 0.5 nm) compared with the broad distribution (at 2 nm with 1-nm width) in the monomeric state representing an extended and stabilized structure in the complex with the N-domain of cTnC. The results demonstrated that the 23/27 region is a mixture or equilibrium between a partial (short) and transient α-helix-like structure and a somehow extended and stabilized conformation in the complex state. In other words, the residues including 23/27 are partially folded to a stable extended conformation upon being fixed stereospecifically on the cTnC surface although still taking a partial (short and transient) α-helix-like structure.

In the EPR spectrum of 43/47, it is evident that the conformation of the α-helix for a short central distance (0.8 nm) with much narrower width (0.67 nm) was exclusively predominant compared to other segments from the predicted IDR as well as its monomeric state (1.5–2.0 nm width) (Fig. 4e*, middle panels, red lines;* Table 2). We could also analyze the data by using 2 Gaussian distributions with ~3 times better χ^2^ (Fig. 4e, *right panel, red line*; Table 2). A large fraction (48%) showed a very narrow distribution centered at 0.9 nm with a 0.1-nm width representing an α-helical structure, and only a small fraction (29%) had a relatively broad distribution ranging from 1–1.8 nm (centered at 1.2 nm with a 1.3-nm width), representing a partial folding or a melted helix conformation in the complex with the N-domain of cTnC. The region 43/47 was represented as an α-helix in the crystal structure of the complex (Fig. 2). The distance observed for 43/47 as very short, and narrow distribution centered at 0.9 nm with a 0.1-nm width is a good natural control for the α-helix in our system and is expected from the previous EPR measurements on a model α-helical peptide^28,29^. At lower ionic strength, we uncovered that for 23/27 and 43/47, which contain PKA and PKC phosphorylation sites, there was an evident transition from flexible in the monomer to a comparably stable conformation in the complex, suggesting a significant function for these residues, either by direct binding to cTnC or by regulating the binding kinetics to cTnC by their nearby regions.

### Mobility of spin-labeled side chain in cTnI N-extension and cTnC N-domain

At room temperature, the spin label (Fig. 1) would rotate within some certain limited range due to the extents of the global protein and local backbone rotation as well as the thermodynamics of the spin labels. In 30% w/v sucrose solutions, the main contribution for the residual mobility is derived from the local backbone rotation, restricted to surrounding binding partners, ligands or proteins. Therefore, by comparing designated spin-labeled amino acids on the mid portion including PKA sites of N-extension, it is possible to screen interactive candidates with the cTnC.

In this study, for 0.2 M KCl experiments, the doubly labeled samples used for distance measurements were examined at room temperature. Only residues 43 and 47 showed small spectral changes to the slow component upon the complex formation with cTnC whereas the other residues had almost no changes within the experimental error (data not shown). This result indicates that the distal main portion still undergoes fast residual motion upon the complex formation as reported previously for the IDR complex^26^. For 0.1 M KCl experiments, the monoresidues 15, 20, and 27 mutated to cysteine and were spin labeled with MSL (Fig. 1). All of these residues showed very fast nanosecond motion (effective rotational correlation time = ~1.5 ns), supporting the above conclusion that the N-extension of cTnI is intrinsically disordered in the monomer state (Fig. 5a–c). Furthermore, the spin-label spectrum showed a single motional component, and we could not resolve this component into the α-helix-like and unfolded structures that were observed in the distance measurements, indicating two structures interconverting as fast as on a nano- or sub-nanosecond time scale. We demonstrated a weak or dynamic interaction of these residues with cTnC at 0.1 M KCl. For residues 15 and 27, there was an immobilized peak on the lower magnetic field (effective rotational correlation time = 5–10 ns) in the complex (Fig. 5a, c, *red line*) compared with its monomeric state (*blue line*). In fact, the slow spectral components were produced by subtracting 30–40% of the monomeric spectra from the cTnC-bound cTnI spectra (Fig. 5). The residue 27 interacts with cTnC strongly and stereospecifically because residues 23/27 reduce the distance upon the complex formation (Fig. 4b, Table 2). On the other hand, the interaction between residue 15 and cTnC is weak because the residues 15/19 do not change the distance distribution (Fig. 4a, Table 2). The spin label mobility of residue 20 was not reduced upon binding to cTnC because at least this residue was expected to dissociate from cTnC by eliminating a positive charge when arginine 20, which is one of the phosphorylation motifs (20–24th residues), was replaced by the cysteine 20-spin label (Fig. 5b, *red and blue line*s).

It is also possible to screen the interaction sites of the cTnC N-domain with the cTnI N-extension (Fig. 2). First, residue 35 near Ca^2+^ binding site 1 of cTnC was selected because this residue was insensitive to conformational changes upon calcium binding to the cTnC-I complex from the spin label mobility^23^ and upon the complex formation between cTnC and C-terminal cTnI peptide from NMR chemical shift^6,8,16^. The spectra obtained at 0.1 M KCl showed that the immobilized peak on the lower magnetic field (effective rotational correlation time = ~10 ns) was clearly enhanced in the complex (Fig. 5d, *red line*) compared with its monomeric state (*blue line*) where the spectrum mainly had a mobilized peak (effective rotational correlation time = ~1.5 ns). The slow spectral components were produced by subtracting 45% of the monomeric spectrum from the cTnI-bound cTnC spectrum (Fig. 5d, *bottom*). Next, residue 69 near Ca^2+^ binding site 2 of cTnC was selected because this residue resides at the tip of the loop between helix C and D, and its side chain is free from intermolecular conformational changes. The spectrum became broadened (effective rotational correlation time = ~2 ns) in the complex compared with its monomeric state (effective rotational correlation time = ~1.5 ns) (Fig. 5e).

### Relation to other work

There was a mixture or equilibrium of partial folding or completely unfolded main structure and minor α-helix-like structure for the doubly spin-labeled N-extension of cTnI both with and without cTnC. It is interesting that α-helical or α-helix-like structures coexist with the unfolded structure along the entire regions of the N-extension. The NMR characterization indicated considerable heterogeneity but could not detect α-helical structure along entire regions^6–9,14–16^. It is, therefore, likely that this structure will be partially and transiently formed along the extension. The MD simulation^33^ showed a transient and short helix formation and decomposition at a nanosecond time scale where NMR does not resolve a minor (10–20%) helix state from a main unfolded state: if the populations of the fast interconverting species are shifted toward the main species, then direct detection of the minor peak can be difficult or impossible^34^.

At physiological ionic strength (0.2 M KCl), almost no or slight structural change occurs in MSL-labeled N-extension upon the complex formation. These slight or no structural changes are very much consistent with that previously reported for the characterization of spin-labeled IDR during interactions with a target protein^26^. It is accepted that the N-extension binds to cTnC of the complex at a physiological ionic strength. The changes in the quantum yield of the fluorescence label at the residue of the N-extension of cTnI showed that it complexed with cTnC even at 0.3 M KCl^18^. Additionally, the N-extension-dependent regulation of the calcium binding affinity occurs in the actomyosin complex^9,12^ or in muscle fiber^10,11^ at physiological ionic strength. Upon binding their target protein, however, IDRs were assumed to lose most of their conformational freedom and adopt a well-defined structure. This view is not valid. IDPs would interact with their target protein *via* a transiently folded short motif, which would require recognition to function. Consequently, IDRs could retain their disordered state in the functional complex^35^. It is, therefore, concluded that the specific conformational changes of the extension would be hidden due to the short-lived and partial (short) folding at 0.2 M KCl. The long-lived secondary structure of the N-extension would be formed by the stronger interaction with cTnC. The previous fluorescence and calorimetric studies also reported that the binding of the cTnI N-terminal extension to the N-terminal domain of cTnC is severely dependent on the ionic strength^18,31^. We reduced the ionic strength to around half (0.1 M KCl). The distance measurements were performed at higher resolution by using MTSL with a less dynamic rotamer than MSL. There was remarkable immobilization of the main portion of the MSL-labeled N-extension complexed with cTnC at 0.1 M KCl. Furthermore, we found an extended stable structure in addition to an α-helical structure at 23/27 and a totally α-helical structure at 43/47 residues of the MTSL-labeled N-extension in the cTnI-cTnC complex.

We found that the conformation of the α-helix at 43/47 residues in the complex for a central distance (0.8 nm) had less distance width (0.78 nm) in the complex at 0.1 M KCl as expected from the EPR measurement on a model α-helical peptide compared to the monomeric state (1.48 nm). Ward *et al*.^14^ and Hwang *et al*.^16^ reported that binding of cTnC to the 1–64 or 1–73 peptide of cTnI including 43 and 47 induces shifts or disappearances of the NMR signals from the C-terminal region of residues 38–67. This is consistent with this region of cTnI forming an α-helix when it complexes with cTnC.

Howarth *et al*.^15^ used solution NMR to determine the structure of free synthetic peptides (32 residues) of the N-terminus extension at 0.15 M KCl and a pH of 6.8. They found that the ends (residues 6–9 and 25–30) of the N-terminus form two α-helices joined by an extended left-handed poly (L-proline) II helix (residue 10–20) and a flexible peptide (residue 21–24). On the other hand, Ward *et al*.^14,36^ found that circular dichroism of the synthetic N-terminus extension (64 residues) showed no secondary structure in s free state, but the NMR signals from residues 18–37 at 0.02 M KCl and a pH of 7.0 were affected by cTnC binding. Sykes and his colleagues^16^ also performed a more detailed analyses of the structure of synthetic peptides of the N-terminus extension (73 residues) at 0.1 M KCl and a pH of 6.2 and found that the entire region (residues 1–37) had no secondary structure, but residues 15–37 were substantially immobilized upon binding to cTnC. Our results appear to be an intermediate between a rigid structure and no secondary structure from these previous studies. We demonstrated that there is no defined structure but narrow 1–1.5 nm distributions at residues 15/19 and 27/31 of an extended left-handed poly (L-proline) II helix or semifolded structure and a broad distribution of no secondary structure at 23/27 and 31/35 in the monomer state at 0.1 M KCl and a pH of 7.0. There is also 10–20% of a sharp peak at 0.9 nm of an α-helix-like structure along the entire length. Furthermore, the spin label mobility of residues 15 and 27 was reduced and residues 23/27 showed a shortened and narrowed distribution upon binding to cTnC. It is, therefore, suggested that there is equilibrium of an α-helix-like structure and semifolded/unfolding state and that a similar equilibrium occurs between two structures that are immobilized, and the phosphorylation site Ser23/24 is stereospecifically fixed upon the binding of cTnC. The NMR spectrum from the solution at room temperature would not resolve the rapid motion of the structures we detected in the frozen solution by using EPR distance measurements. The differences would also be due to the length of the synthetic peptides that were used instead of a full-length cTnI we used. In fact, there was no measurable increase in the calcium affinity of cTnC upon peptide-cTnC complex formation^16^, although the full-length cTnI increases the affinity markedly^9–12^.

The crosslinking study^19,20^ performed at 0.05–0.1 M KCl/NaCl showed that residues 10, 18, 19 and 26 of the N-extension of cTnI interact with cTnC, especially residue 19 with residues 47 and 80 of cTnC, and residue 5 of cTnI interacts with residues 154 and 155 intramolecularly. Our results showed that when cTnI was in complex at 0.1 M KCl, the distance distribution of the residues 23/27 became narrower, but residues 15/19 were still broad and did not show any discernable change. The spin label mobility of residues 15 and 27 had a slow component when cTnI was complexed with cTnC. It is therefore concluded that only the region including residues 23–27 interacts stereospecifically with cTnC at 0.1 M KCl, although many residues 5, 10, 18, and 19 of the cTnI extension interact weakly with cTnC and cTnI.

Howarth *et al*.^15^ docked their NMR structure at 0.15 M KCl onto a crystal structure to create a static model of the N-extension within the troponin complex, and the results were consistent with the small angle neutron scattering data. Cheng *et al*.^29^ also performed molecular-dynamics (MD) simulations to elucidate the dynamic structure of the N-extension in the cTnC-I-T complex at a 0.15 M ionic strength. In the MD model, the N-extension interacts with the N-lobe of cTnC and becomes stabilized. cTnI sites Ser23/24 contact closely with the B-helix of cTnC. Our results at 0.1 M KCl support this model. Residues 23/27 had a discernable transition from flexible to a relatively stable extended conformation with a central distance (1.4 nm) and a narrow width (0.5 nm) when cTnI is complexed with cTnC. Furthermore, the side-chain spin label of residue 27 of the N-extension is immobilized. Jayasunda *et al*.^37^ also performed MD simulations to elucidate the dynamic structure of N-extension in the cTnC-I-T complex at 0.15 M NaCl using fluorescence energy transfer distances as restraints. In this model, the N-extension interacts with the N-lobe of residues 31–33 of cTnC:cTnI to form a hydrogen bond with the residues at site 1 and site 2 of cTnC. However, the distance distributions of residues 27/31 and 35/39 near residues 31–33 remained broad and showed almost no discernable change when cTnI was complexed at 0.1 M KCl.

The interaction sites of cTnC with the N-extension IDR of cTnC have been studied using NMR in a 0.15 M KCl solution^6,7^. Site 1 and site 2 were proposed for the interaction sites. The MD simulation also showed that the N-extension interacts with the residues at site 1 and site 2 of cTnC at a 0.15 M ionic strength^38^. Our results at 0.1 M KCl support this model. The spin labels at residue 35 at site 1 and residue 69 at site 2 were immobilized.

## Conclusions

This is the first report of the successful measurement of the distance between two cysteine residues, specifically 15/19, 23/27, 27/31, 35/39, and 43/47, of the N-extension of cTnI by using site-directed spin labeling EPR spectroscopy to predict the secondary structure. At 0.2 M KCl, a highly flexible or unstructured conformation of N-extension emerged in both the cTnI-cTnC complex and the monomer state. From mobile side-chain residues and main broad residual distance distribution over 1–2 nm with a minor sharp peak at 0.9 nm, we predicted unfolded structures including a transient and partial α-helix-like structure. The specific secondary structures would be hidden at a physiological ionic strength of 0.2 M and could be uncovered and resolved by lowering the ionic strength. At 0.1 M KCl, the distance distributions of only two pairs of residues, 23/27 and 43/47, were remarkably narrower in the cTnI-cTnI complex than in the monomeric state. The 23/27 and 43/47 regions have PKA and PKC phosphorylation sites and form a stable extended and α-helical structures in the complex state, respectively. Residues 15 and 27 were partially immobilized by complex formation. We propose that these regions are the main determinants of the binding of the N-extension to cTnC and that the binding is quite dynamic especially at 0.2 M KCl. The N-extension immobilizes the residues at site 1 and 2 of cTnC. Then, the 23–27 regions of cTnI bind to cTnC, which stabilizes the conformation with high Ca^2+^ affinity. Phosphorylation of serine 23 and 24 abolishes this interaction, having an effect similar to truncation or removal of the N-extension of cTnI^9–12^. It is possible that upon phosphorylation, the introduction of negative charges and localized conformational changes within the N-terminal extension of cTnI reduces its affinity for cTnC, thus relieving the stabilization and enhancing Ca^2+^ release. Similarly, the 43–47 region responds to PKC phosphorylation of serine 42 and 44 as does the 23–27 region. The present study provides an opportunity for future research on phosphorylated states or phosphomimic mutations and adds to the work aimed at searching for the binding sites on cTnC by intermolecular distance measurement. These studies will be useful for understanding and treating diseases such as hypertrophic cardiomyopathy.

## Methods

### Plasmids and mutagenesis

Original pET-15b-cTnC (anti-ampicillin), cysteine-less cTnC plasmid and cysteine-less cTnI plasmid were gifted from Dr. Tatsuhito Matsuo (JAEA) or provided by Dr. Shoji Ueki, one of the authors, both of which originated from Dr. Yuichiro Maeda (Nagoya University). We introduced two cysteines at A15 and I19 on the cardiac N-terminal extension of cTnI (cTnI1519) as C80S/C97S/A15C/I19C and prepared from a template a cysteine-less mutant (C80S/C97S). Similarly, we prepared cTnI2327, cTnI2731, cTnI3539, and cTnI4347 as C80S/C97S/S23C/R27C, C80S/C97S/R27C/T31C, C80S/C97S/A35C/S39C, and C80S/C97S/A43C/L47C, respectively.

### Expression and purification

cTnC and cTnI mutants were expressed and purified as previously described^22^. Briefly, mutant plasmids were used to transform competent *Escherichia coli* BL21 Star (DE3) One Shot cells. Protein production was induced through the addition of 1 mM ß-thiogalactoside, and induction was allowed to proceed for 6 h (cTnC) or 13 h (cTnI). The cells were harvested by centrifugation, and then, the cell pellet was suspended in a buffer solution containing 10 mM Tris and 1 mM EDTA (pH 7.8), sonicated. ammonium sulfate (60% w/v) was added to the cell extract in the buffer, and the solution was centrifuged. The supernatant was then dialyzed, loaded onto a Q-Sepharose anion exchange column (Amersham/GE Healthcare, Piscataway, NJ) equilibrated with the same buffer, and eluted with a KCl gradient; the protein purity was confirmed by SDS-PAGE. Protein concentrations were determined by a BCA protein assay (Thermo Fisher Scientific, Rockford, IL) with BSA as the standard. Dithiothreitol (DTT) (2 mM) was added to the protein solution, which was then stored at −80 °C.

Human cardiac TnI mutants were expressed using the same method for cTnC. The cell pellet was suspended in a buffer solution containing 20 mM Tris, 8% sucrose, 5% Triton X-100, and 5 mM EDTA (pH 7.5), was incubated at room temperature for 1 h, and was then sonicated. The sonication procedure was repeated twice in the same buffer and once in 20 mM Tris and 1 mM EDTA (pH 7.5). The pellet was then resuspended in 6 M urea, 20 mM Tris, 0.1 M KCl, and 1 mM EDTA (pH 7.5) and centrifuged. The supernatant was applied to an SP Sepharose or CM Sephadex cation exchange column (Amersham/GE Healthcare) equilibrated with the same buffer and eluted with a KCl gradient. The protein purity and concentration were determined as described above.

### Spin labeling and preparation of a TnC-TnI binary complex

We used the 4-maleimido-2,2,6,6-tetramethyl-1-piperidinyloxy (MSL) and (1-oxyl-2,2,5,5-tetramethylpyrrolidin-3-yl) methyl methanethiosulfonate (MTSL) spin-labels purchased from Sigma-Aldrich (St. Louis, MO) and Toronto Research Chemicals (North York, ON), respectively. After removal of DTT from the stock solution by application to a Sephadex G-25 desalting column (Amersham/GE Healthcare) equilibrated with a buffer containing 20 mM MOPS, 0.2 M KCl, and 1 mM EDTA (pH 7.1) and 6 M urea, the cTnI mutant was incubated with a 10-fold molar excess of MSL or MTSL. The reaction was allowed to proceed at room temperature for 1 h followed by incubating the mixture overnight at 4 °C. Unattached MSL or MTSL was removed by application to a Sephadex G-25 desalting column and by dialysis against the same buffer to completely remove free spin label. The spin-label:protein ratios estimated by double integration of EPR spectra were 1.8:1. Spin-labeled cTnC was prepared as described by Ueki *et al*.^22^.

A human cardiac cTnC-cTnI binary complex was prepared as described by Abe *et al*.^39^ and Ueki *et al*.^22^. Briefly, cTnC and spin-labeled mutant TnI were mixed (1:1.5) in a buffer containing 20 mM MOPS, 6 M urea, 1 M KCl, and 50 mM Ca^2+^ (pH 7.1), and then stepwise dialyses were carried out to reduce the urea concentration. The final buffer solution contained 20 mM MOPS, 0.2 M KCl, 3 mM MgCl2, and 1 mM EGTA (pH 7.0). cTnI that did not form a complex with cTnC was removed by centrifugation. The formation of a binary complex was confirmed by urea-PAGE^40^.

### EPR spectroscopy and analysis

CW (continuous wave)-EPR measurements were performed with a Bruker ELEXSYS E500 spectrometer (Bruker Biospin, Yokohama, Japan) as described previously^22–24,41,42^. For residual mobility measurement, either the cTnI monomer or the cTnI-cTnC complex labeled with MSL or MTSL was dialyzed against buffer containing 20 mM MOPS, 0.1 M KCl, and 1 mM EGTA; 5 mM CaCl_2_ was supplemented for low ionic strength conditions. For high ionic strength, 0.2 M KCl and 3 mM CaCl_2_ were supplemented, then 30% sucrose was added, and the mixture was held on ice for 1 h to inhibit interference due to the global movement of the protein. Samples (15 μL) were loaded into 1.0 mm outside diameter capillaries sealed on both ends. EPR spectra were acquired using 1 G field modulation amplitude at 100 kHz and a 5 mW incident microwave power at 295 K with a central magnetic field of 3440 G, a microwave of ~9.65 GHz, and a magnetic field intensity range of 100 G. For distance measurement, samples (100 μL) were loaded and measured at 170 K with a 0.1 mW incident microwave power, a central magnetic field of 3430 G, a microwave of ~9.6 GHz, and magnetic field intensity range of 200 G.

The effective rotational correlation time was calculated according to the equation given by Goldman *et al*.^43^: τ_eff_ = a(1 − T_eff_/T_max_)^b^, where a = 5.4 × 10^−10^ s, b = −1.36, T_max_ = 35. G is the rigid limit for a particular spin, and 2T_eff_ is the effective splitting between the low-field and high-field absorption peaks. Assuming that the experimental spectrum contains only two single components corresponding to the fast and slow motion of the spin label, we estimated the ratio of the peak height between the slow and fast components at a lower magnetic field as an indicator of steric hindrance around the side chains.

The dipolar CW-EPR method has been shown to provide accurate distributions of the distances between spin labels in the range from 0.8 to 2.5 nm^22,27–29,32,44–47^. To eliminate broadening by the motional effect, the double-labeled spectrum was obtained in a frozen solution at 170 K. The dipolar spectrum is a convolution of the spectrum with no spin-spin interactions (single-labeled spectrum) with a broadening function. The spectrum was fit by changing 3 or 6 parameters: the center (*r* = 0.8–2.8 nm) and full-width (*Δr* = 0.04–2 nm) at half maximum of the distance distribution between the spin labels (modeled as single or double Gaussians) and the percentage fraction (*f* = 0–100%) of the Gaussian population or noninteracting spins^32,44–46^. Spectral broadening of the double-labeled samples was analyzed using a Levenberg-Marquardt algorithm in software written in Igor Pro (Wavematrix, Inc, Lake Oswego, OR) developed in our laboratory^27,46,47^ and a Monte Carlo/Simplex Gaussian convolution method in software CWdipFit^32,45^ running on the MATLAB platform.
